# Cestodes in Eurasian wolves (*Canis lupus lupus*) and domestic dogs (*Canis lupus familiaris*) in Switzerland

**DOI:** 10.1016/j.ijppaw.2024.101027

**Published:** 2024-11-30

**Authors:** Anna Schneider, Gastón Moré, Mirjam Pewsner, Caroline F. Frey, Walter Basso

**Affiliations:** a Institute of Parasitology, Vetsuisse Faculty, University of Bern, Länggassstrasse 122, 3012, Bern, Switzerland; b Institute for Fish and Wildlife Health, Vetsuisse Faculty, University of Bern, Länggassstrasse 122, 3012, Bern, Switzerland

**Keywords:** *Taenia*, *Echinococcus*, Multiplex-PCR, DNA-Sequencing, Flotation

## Abstract

Eurasian wolves (*Canis lupus lupus*) and domestic dogs (*Canis lupus familiaris*) are definitive hosts of numerous cestode species. While infections with adult stages in canids are usually subclinical, some species pose a zoonotic risk or cause infections in wildlife and livestock, resulting in disease and/or economic losses. This study aimed to determine the prevalence, species composition, and geographical distribution of cestode infections in dogs and free-ranging wolves in Switzerland. Faecal samples from 2065 dogs and intestinal content from 121 necropsied wolves were macroscopically examined and tested using zinc chloride flotation method. When cestode eggs or adult cestodes were detected, a molecular identification based on multiplex-PCR and sequencing was performed. In the sampled wolves, the prevalence by flotation (42/121; 34.7%) was lower than the overall prevalence including macroscopic examination (76/121; 62.8%). The flotation method thus failed to detect cestode infections in 44.7% (34/76) of infected wolves. The most frequently detected species was *Taenia hydatigena* (46/121; 38.0%), followed by *Taenia serialis* (23/121; 19.0%), *Mesocestoides* spp. (3/121; 2.5%), *Taenia ovis* (1/121; 0.8%), and *Echinococcus multilocularis* (1/121; 0.8%). In the analysed dogs, the prevalence was 0.9% (19/2065), but the real prevalence is very likely to be higher, as no necropsy data were available. Identified cestode species included *Taenia crassiceps* (6/2065; 0.3%), *E. multilocularis* (3/2065; 0.1%), *Mesocestoides* sp. (2/2065; 0.1%), *Taenia polyacantha* (1/2065; 0.05%), and *Dibothriocephalus latus* (1/2065; 0.05%). By identifying the cestode species infecting two closely related host species with markedly different lifestyles, this study sheds light on the local distribution of these parasites and their potential impacts on wildlife, livestock, and human health. Due to their close contact with humans, infected dogs represent an important source of infection with zoonotic cestodes such as *Echinococcus* spp. and certain *Taenia* species, responsible for serious human diseases.

## Highlights

• 62.8% of the wolves were infected with cestodes.
• The flotation method failed to detect 44.7% of cestode infections in wolves.
• 38.0% wolves were infected with *T. hydatigena*, 19.0% with *T. serialis*.
• Prevalence for a cestode infection in dogs was 0.9%.
• Dogs carried zoonotic species such as *T. crassiceps* or *E. multilocularis*.

## Graphical abstract

**Figure:**
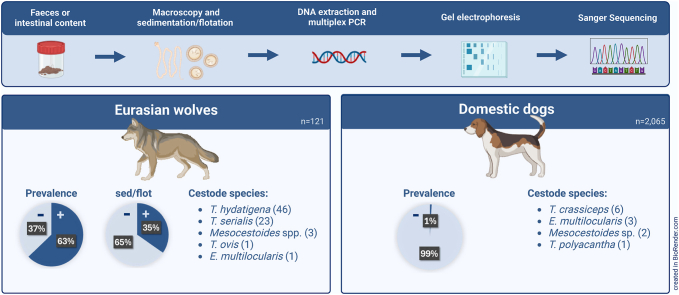

## 1 Introduction

Domestic and wild canids serve as definitive hosts for many cestode species. The family Taeniidae, particularly the genera *Taenia* and *Echinococcus*, includes species of great importance in both veterinary and human medicine (Hiepe et al., 2006; Deplazes et al., 2021; Gunn and Pitt, 2022). Cestodes have heteroxenous life cycles, with both their adult and larval (metacestode) stages being parasitic. Adult cestodes are typically localized in the small intestine of their definitive hosts, and infections are usually subclinical. However, in some cases, clinical signs such as weight loss, anorexia, mild gastrointestinal signs or a poor hair coat may occur. Additionally, infections with certain *Taenia* species and *Dipylidium caninum* can cause anal pruritus, caused by free motile proglottids, which may be shed independently of defecation (Deplazes et al., 2021; Rousseau et al., 2022). In contrast, the larval stages infect various organs, tissues, and body cavities of a wide range of intermediate hosts, including both wild and domestic animals, as well as humans, and are often associated with clinical manifestations. In livestock, these infections can cause considerable economic losses (Deplazes et al., 2021; Gunn and Pitt, 2022). The larval stages of *Echinococcus* spp. can lead to serious diseases in humans and animals, such as alveolar echinococcosis (AE) (caused by *E. multilocularis*) and cystic echinococcosis (CE) (caused by *E. granulosus*) (Corsini et al., 2015; Gottstein et al., 2015; Deplazes et al., 2021). Notably, AE has been ranked first as a priority infection among food-borne parasites in Europe (Bouwknegt et al., 2018).

In addition, larval stages of *Taenia* spp. of canids can sometimes cause disease in humans, such as cysticercosis due to *T. crassiceps*, coenurosis due to *T. multiceps* and *T. serialis*, or sparganosis due to *Spirometra* spp. (Deplazes et al., 2019). Interestingly, besides being definitive hosts, canids may occasionally act as aberrant intermediate hosts of several cestode species such as *E*. *multilocularis, T. crassiceps, Spirometra* spp. and *Mesocestoides* spp. The metacestodes affect mainly the liver, body cavities or subcutaneous tissues, with variable clinical manifestations such as hepatitis, peritonitis, ascites, subcutaneous swelling, or massive metacestode proliferation with fatal outcome (Drake et al., 2008; Corsini et al., 2015; Carta et al., 2021; Murphy et al., 2021).

In Switzerland, the Eurasian wolf (*Canis lupus lupus*) was eradicated by the end of the 19th century. The first wolves migrated back to Switzerland in 1995 and formed the first packs in 2012. Since then, several Swiss and transboundary packs have formed with approximately 300 detected individuals (Graf and Fischer, 2023; KORA - Raubtierökologie und Wildtiermanagement, https://www.kora.ch/de/, accessed 6th June 2024). All wolves found dead or culled due to health-related issues or management interventions were submitted to the Institute for Fish and Wildlife Health (FIWI) at the Vetsuisse Faculty in Bern for post-mortem examination and collection of baseline health data (Swiss Federal Office of Environment, 2016).

According to AMICUS (https://www.amicus.ch, accessed 18th July 2024), the national dog registration database in Switzerland, the number of registered dogs (*Canis lupus familiaris*) has grown from 497,334 in January 2017 to 553,324 in May 2024. These data indicate that while the population of wolves is increasing, the growth in the number of domestic dogs is even more important. Dogs and wolves, although widely differing in their lifestyles, may be hosts to the same cestode species. While domestic dogs are usually fed and receive veterinary care, including parasite control, free-ranging wolves must hunt for food and are never treated for infections. They may therefore reflect the cestode species circulating in their habitats much more accurately than the domestic dogs. Dogs, on the other hand, may pose a greater risk of metacestode infection to livestock and humans because they live in much closer contact and heavily outnumber free-ranging wolves.

Diagnosis of infection in definitive hosts as well as identification to species level are important to establish appropriate control measures to minimise the possible zoonotic and economic consequences. The diagnosis of intestinal infection in carnivores is mainly achieved by coproscopical methods to detect proglottids or worm eggs, and also by inspection of the perineal region for proglottids adhered to the fur (Deplazes et al., 2021). However, eggs of different *Taenia* spp. are morphologically indistinguishable among them and also from those of *Echinococcus* spp. Therefore, molecular tests must be performed for further identification at genus and species level (Trachsel et al., 2007; Deplazes et al., 2021). In addition, coproantigen detection methods were developed for some cestodes such as *Echinococcus* spp. and *Dipylidium caninum* (Deplazes et al., 1999; Christofi et al., 2002; Conraths and Deplazes, 2015; Little et al., 2023).

The objectives of this study were to assess the prevalence of cestode infections, the occurring cestode species and their geographical distribution in domestic dogs and free-ranging wolves in Switzerland by parasitological and molecular methods. Additionally, the potential economic and zoonotic impacts of the detected cestode species are discussed.

## 2 Material and methods

### 2.1 Study population

To determine the presence of cestodes in canids in Switzerland, samples and data from free-ranging Eurasian wolves (hereafter referred to as “wolves”) and domestic dogs were collected between January 2017 and May 2024. Over this period, a total of 121 wolves and 2065 dogs were examined coproscopically at the Institute of Parasitology in Bern (IPB) and were included in this study. All wolves in the study were found dead or culled and were submitted to the FIWI for post-mortem examination and the coordinates of their location were documented for 118/121 wolves. Dog faecal samples were received at a daily basis for routine analysis at IPB during the study period. The dog samples were localized using the owner's postcode, which was documented for 2049/2065 samples.

### 2.2 Necropsy performed on free-ranging Eurasian wolves

Post-mortem examination was performed on all 121 wolves. This examination included a gross necropsy, performed according to a standard protocol (Ryser-Degiorgis and Segner, 2015). Body cavities were opened, and organs systematically removed, weighed, cut open and macroscopically assessed. The gastrointestinal tract was opened along most of its length, except for small portions that were left intact for further sampling for histological examination, which was not part of this study. Intestinal contents, mainly from the rectum, including adult helminths were collected and further examined at the IPB.

### 2.3 Parasitological and molecular diagnostics

Intestinal contents collected during necropsy (121/121 wolf samples, 9/2065 dog samples) or faecal samples (2056/2065 dog samples) were macroscopically examined for adult worm stages. and tested for cestode eggs using a sedimentation/flotation technique (44% zinc chloride solution) (in the following “flotation”), as described by Deplazes et al. (2021). Flotation was performed on all 121/121 wolf samples and on 2063/2065 dog samples, as two of these samples consisted almost entirely of worm stages with only a small amount of faeces. A total of 127/2065 samples from dogs were additionally analysed by sedimentation, described by Deplazes et al. (2021) as well. This method was not used as standard in the analysis of dog faeces, but only at special request of the submitter. For wolf samples, intestinal contents were frozen at −80 °C for 72 h prior to analysis to inactivate potentially present *Echinococcus* spp. eggs to reduce the risk of accidental infections in the lab. Adult parasites, as well as putative proglottids present in the faeces or intestinal content were microscopically identified up to genus level. For further identification to the species level, DNA was extracted from faeces/intestinal content in which cestode eggs were microscopically detected, as well as from proglottids using commercial kits (i.e., Quick-DNA Fecal/Soil Microbe Miniprep Kit, Zymo Research Corporation, USA and DNeasy Blood & Tissue Kit, QIAGEN GmbH, Hilden, Germany, respectively) according to the manufacturer's instructions.

A multiplex PCR was performed to identify the morphologically indistinguishable taeniid eggs, using three pairs of primers (Trachsel et al., 2007). These primers target the *nad 1* gene for both *E. multilocularis* (395 bp) and *E. granulosus* (117 bp), as well as the *12S rRNA* for *Taenia* spp., *Hydatigera* spp., *Mesocestoides* spp., *Dibothriocephalus* (syn. *Diphyllobothrium*) spp. and *Dipylidium* spp. (267 bp). For each PCR run, DNA from *E. multilocularis*, *E. granulosus* and *T. saginata* were used as positive controls, while sterile water served as a non-template control. For the PCR of *Taenia*/*Mesocestoides* proglottids, only the two primers targeting the *12S rRNA* gene fragment were utilized, as *Echinococcus* spp. had been excluded based on morphological examination (Trachsel et al., 2007). The amplified products were visualized using 1.5% agarose gel electrophoresis stained with ethidium bromide.

The PCR products were purified and concentrated using the DNA Clean & Concentrator-5 kit (Zymo Research Corporation, USA). For DNA samples positive for *Taenia* spp. and *Echinococcus* spp., amplification products were extracted from the agarose gel and purified using the Zymoclean Gel DNA Recovery Kit (Zymo Research Corporation, USA). These purified products were then submitted to Microsynth AG (Balgach, Switzerland) for bidirectional Sanger sequencing using the same primers used for PCR amplification.

Sequence analysis and alignment were performed using the software Geneious Prime v2024.0.5 (Biomatters Ltd., Auckland, New Zealand). The resulting consensus sequences were trimmed from the primer regions and compared to sequences in GenBank® using the Basic Local Alignment Search Tool (BLAST, https://blast.ncbi.nlm.nih.gov/Blast.cgi).

### 2.4 Statistical analysis

Microsoft Excel 365 for Windows (Microsoft Corporation, Redmond, USA) was used to calculate percentages, maximums and minimums. The 95% confidence intervals (95% CI) were calculated using the Sample Size Calculators for designing clinical research website (Kohn and Senyak, 2021). Statistical analyses (Chi-squared test) were performed using R Studio 2023.06.1 (https://www.rstudio.com/). Mapping was conducted with the Geographic Information System QGIS 3.34 (https://www.qgis.org).

## 3 Results

### 3.1 Samples from free-ranging Eurasian wolves

The wolves were categorized by sex as follows: 41 females (33.9%), 78 males (64.5%), and two with unknown sex (1.7%). Overall, 76 samples (62.8%) tested positive for a cestode infection. The prevalence of an infection with cestodes was 61.0% in females and 65.4% in males which is not statistically significant different (*p* = 0.63). Table 1 shows the distribution of cestodes positive wolves according to age categories.

**Table 1:** Age group structure of sampled wolves (n = 121) and wolves in which cestodes were detected (n = 76).

Age group	n	%	n positive	% positive	95% CI
Juvenile (<1 year old)	42	34.7	25	59.5	43.3–74.4
Adult (≥1 year old)	76	62.8	50	65.8	54.0–76.3
Unknown age	3	2.5	1	33.3	0.8–90.6

Total	121	100	76	62.8	53.6–71.4

The samples were classified into four categories regarding results from flotation and adult stages observation at necropsy and/or macroscopic examination of the sample: (i) positive by flotation and detection of adult stages (35/121; 28.9%), (ii) positive by flotation but no detection of adult stages (7/121; 5.8%), (iii) negative by flotation but adult stages observed (34/121; 28.1%) and (iv) negative for cestodes in flotation and no detection of adult stages (45/121; 37.2%). Therefore, the overall prevalence for an infection with cestodes was 62.8% (76/121; 95% CI: 53.6–71.4%) but the prevalence based solely on flotation was 34.7% (42/121; 95% CI: 26.3–43.9%).

Cestode identification at the species level was performed by PCR and sequencing on 72/76 positive samples using DNA extracted from adult stages (n = 66) or faeces (n = 6). Table 2 shows the detected cestode species including macroscopic identification of adult stages and flotation results. Four positive samples were only characterized at the genus level as *Taenia* sp., either through morphological identification of adult stages (3/4) or multiplex PCR on DNA from faecal taeniid eggs (1/4). Sequencing was performed on the sample with DNA extracted from faecal taeniid eggs but resulted in superimposed signals which may be caused by a mixed infection with several *Taenia* spp. or other cestodes. In addition, 41 out of the 42 faecal samples positive for taeniid eggs by flotation were checked for co-infections by PCR and revealed a co-infection with *T. hydatigena* and *E. multilocularis* in one wolf. Noteworthy, only 25 out of 46 wolves infected with *T. hydatigena* showed positive flotation results. All three animals with *Mesocestoides* spp. infection showed negative flotation results as well (Table 2). There was no difference in the occurrence of the cestode species in relation to the wolves age or sex. Negative flotation results occurred with similar frequency in both juvenile (12/25; 48.0%) and adult (22/50; 44.0%) wolves.

**Table 2:** Prevalence (%) of cestode species in sampled wolves (n = 121) by macroscopical and microscopical methods.

Cestode species	Adult stages	%a (n = 121)	Positive flotation	%f (n = 121)	Positive total_1_	% (n = 121)
*T. hydatigena*	46	38.0	25	20.7	46	38.0
*T. serialis*	18	14.9	15	12.4	23	19.0
*T. ovis*	0	0	1	0.8	1	0.8
*E. multilocularis*_2_	0	0	1	0.8	1	0.8
*M. canislagopodis*	2	1.7	0	0	2	1.7
*Mesocestoides* sp._2_	1	0.8	0	0	1	0.8
*Taenia* sp._3_	3	2.5	1	0.8	4	3.3

Total_4_	69	57.0	42	34.7	76	62.8

1Macroscopical detection and/or detection by flotation; 2Coinfection with *T. hydatigena*; 3Morphological identification of adult worms (3/4) or genetic identification of taeniid eggs (1/4) only up to genus level; 4Wolves samples positive for cestodes. Ref: %a = average of adult stages detected for each cestode species; %f = average of positive flotation for each cestode species.

Forty-three of the 46 obtained sequences of *T. hydatigena* were all identical among them and showed 100% identity with *T. hydatigena* GenBank® sequences from a wolf (OQ924353), roe deer (*Capreolus capreolus*, OQ916366) and wild boar (*Sus scrofa*, OQ916367) from Italy, as well as sheep from Iraq (MK858250) and Egypt (LC500214). One of these identical sequences and two further sequences, showing a single nucleotide polymorphism (SNP) with respect to the previous ones, were submitted to GenBank® (accession no. PP978719, PP978720 and PQ309039). Twenty-three sequences showed 100% identity with sequences of *T. serialis*, and one of them was submitted to GenBank® (PP978721). They were identical to *T. serialis* sequences from dogs from Germany (EU219546) and Sweden (KP127677), a roe deer from Italy (OQ916386) and a golden jackal (*Canis aureus*) from Croatia (MF495483). One sequence (PP978723) had a 100% identity with *T. ovis* sequences from New Zealand (NC_021,138) and Australia (KJ591568, query cover 89%) and a 99.50% identity with a sequence from Switzerland (DQ408421, query cover 90%). Two further identical sequences (PP978726) had a 99.55% identity with sequences of *M. canislagopodis* from an arctic fox (*Vulpes lagopus*, KT232152) and a ptarmigan (*Lagopus muta*, KT232151) from Iceland.

**Fig. 1:**
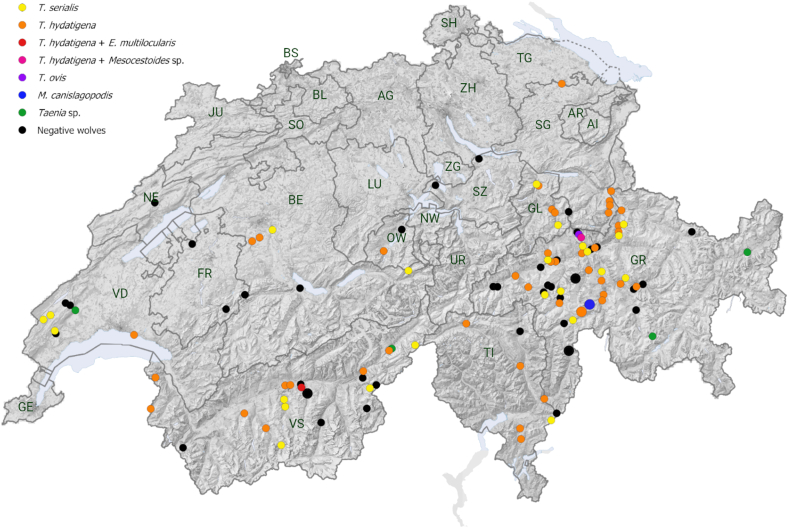
Map of Switzerland with the geographical distribution of cestode positive (n = 76) and negative (n = 42) samples of wolves including the detected cestode species shown as coloured points. Clusters of two wolves from the same location are shown as a bigger marking. Cantons of Switzerland are indicated by two letters as follows: AG = Aargau; AI = Appenzell Inner-Rhodes; AR = Appenzell Outer-Rhodes; BE=Bern; BL=Basel District; BS=Basel; FR=Fribourg; GE = Geneva; GL = Glarus; GR = Grisons; JU = Jura; LU=Lucerne; NE=Neuchâtel; NW=Nidwalden; OW=Obwalden; SG=St. Gallen; SH=Schaffhausen; SO=Solothurn; SZ=Schwyz; TG = Thurgau; TI=Ticino; UR=Uri; VD=Vaud; VS=Valais; ZG = Zug; ZH = Zurich. Cantonal borders are indicated by grey lines.

The geographical distribution of all tested wolf samples and the identified cestode species in Switzerland is shown in Fig. 1. Most of the samples came from the cantons of Grisons (57/121; 47.1%) and Valais (25/121; 20.7%). Three cestode negative tested wolves, from Valais (n = 2) and Grisons (n = 1), are not shown on the map due to unrecorded coordinates. There was no obvious geographical pattern in the distribution of different cestode species.

### 3.2 Samples from domestic dogs

A total of 2065 dogs were tested for cestodes, of which 1023 were male (49.5%), 885 female (42.9%) and 157 with unknown sex (7.6%). Table 3 shows the distribution of cestodes positive dogs according to age categories. Overall, 19 dogs tested positive for a cestode infection, resulting in a prevalence of 0.9% (95% CI: 0.6–1.5%). The prevalence was significantly higher in females (1.5%) compared to males (0.5%) (*p* = 0.027) and tended to increase with age, being highest in senior dogs with 1.8% (Table 3).

**Table 3:** Age group structure of analysed dogs (n = 2065) and dogs in which cestodes were detected (n = 19).

Age group	n	%	n positive	% positive	95% CI
Juvenile (<1 year)	496	24.0	0	0	0.0–0.7
Adult (1–7 years)	891	43.1	7	0.8	0.3–1.6
Senior (≥8 years)	497	24.1	9	1.8	0.8–3.4
Unknown age	181	8.8	3	1.7	0.3–4.8

Total	2065	100	19	0.9	0.6–1.4

The samples were classified into five categories regarding results from flotation and adult stages observed by macroscopic examination: (i) positive by flotation and adult stages detected (1/2065; 0.05%), (ii) positive by flotation and no adult stages found (15/2065; 0.7%), (iii) negative by flotation but adult stages observed (1/2065; 0.05%), (iv) worm stages found in faeces without any flotation carried out (2/2065; 0.1%), and (v) negative for cestodes by flotation and no adult stages (2046/2065; 99.1%). Therefore, the prevalence by flotation alone was 0.8% (95% CI: 0.4–1.3%), while the overall prevalence for a cestode infection was 0.9% (95% CI: 0.6–1.4%).

The detected cestode species and prevalences in the analysed dogs are shown in Table 4. Out of the 19 positive samples, nine were successfully sequenced and characterized at the species level using DNA extracted from either adult worms (n = 2) or faeces (n = 7). Seven samples were positive by flotation but contained only few taeniid eggs and therefore not enough DNA for PCR and sequencing. In one of two dogs infected with *Mesocestoides* sp., flotation was negative, and the adult worms were not stored for DNA extraction. For the second dog, flotation was not carried out as the faecal amount was insufficient and DNA was extracted from proglottids. In the dog infected with *D. latus*, adult worm stages were sent for identification and DNA extraction was performed (Table 4).

**Table 4:** Prevalence (%) of cestode species in faeces from domestic dogs (n = 2065) by macroscopical and microscopical methods.

Cestode species	Adult stages	%a (n = 2065)	Positive flotation	%f (n = 2063)	Positive total_1_	% (n = 2065)
*T. crassiceps*_2_	1	0.05	6	0.3	6	0.3
*E. multilocularis*_2_	0	0	3	0.1	3	0.1
*T. polyacantha*	0	0	1	0.05	1	0.05
*D. latus*_3_	1	0.05	nd	nd	1	0.05_4_
*Mesocestoides* sp._5_	2	0.1	0	0	2	0.1
*Taeniid* eggs_6_	0	0	7	0.3	7	0.3

Total_7_	4	0.2	16	0.8	19	0.9

1Macroscopical detection and/or detection by flotation; 2one coinfection of *T. crassiceps* and *E. multilocularis*; 3only proglottids sent for identification; 4 the real prevalence may be underestimated because the flotation method is not adequate to detect *D. latus* eggs; 5 flotation has only been carried out for 1/2 samples; 6not further identified; 7dog samples positive for cestodes. Ref: %a = average of adult stages detected for each cestode species; %f = average of positive flotation for each cestode species; nd = not done.

Six sequences showed 100% identity to each other and to *T. crassiceps* sequences from red foxes (*Vulpes vulpes*) from Poland (MN505206) and Italy (OQ913861), among others. One of these sequences was submitted to GenBank® (PP978722). One further sequence (PP978724) matched 100% with *T. polyacantha* sequences from a red fox (LT635750) and a bank vole (*Myodes glareolus*, LT635753) from Sweden. The *Mesocestoides* sequence (PP978728) obtained from proglottids had a 99.55% identity to numerous *M. litteratus* GenBank® sequences (MN505210, *Lynx lynx*, Poland; MH992712, *Canis lupus familiaris*, Germany; among others) and also to one sequence from a dog with canine peritoneal larval cestodosis, that was submitted as *M. lineatus* (EF567417, Germany). Therefore, it could not be clearly identified at species level. The obtained sequence from *Dibothriocephalus latus* (PP978725) had identities of 100% with a sequence from *D. latus* from Switzerland (AP017663) and 99% with sequences from Japan (AB269325) and Korea (NC_008945).

**Fig. 2:**
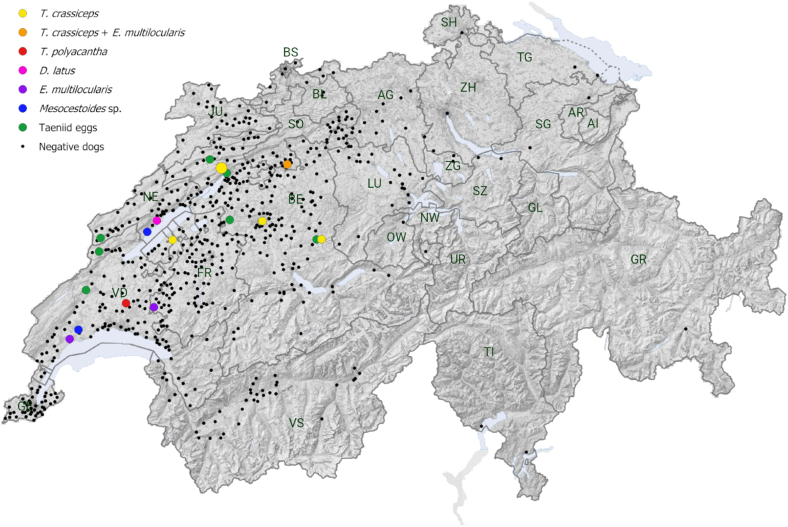
Map of Switzerland with the geographical distribution of cestode positive (n = 19) and negative (n = 2030) samples of dogs including the detected cestode species shown as coloured points. A cluster of two positive dogs is shown as a bigger marking. Clusters in negative dogs are not considered. Cantons of Switzerland are indicated by two letters as follows: AG = Aargau; AI = Appenzell Inner-Rhodes; AR = Appenzell Outer-Rhodes; BE=Bern; BL=Basel District; BS=Basel; FR=Fribourg; GE = Geneva; GL = Glarus; GR = Grisons; JU = Jura; LU=Lucerne; NE=Neuchâtel; NW=Nidwalden; OW=Obwalden; SG=St. Gallen; SH=Schaffhausen; SO=Solothurn; SZ=Schwyz; TG = Thurgau; TI=Ticino; UR=Uri; VD=Vaud; VS=Valais; ZG = Zug; Cantonal borders are indicated by grey lines.

The geographical distribution of all tested dogs and those in which cestodes were detected is illustrated in Fig. 2, shown as coloured points. The distribution of dog samples was predominantly in western Switzerland. Information about the location of 16 negative dogs was not available and are therefore not included on the map. The distribution of the different cestode species did not show a specific geographical pattern.

## 4 Discussion

In Switzerland, all deceased wolves - whether due to natural causes, health- or management-related culling, have been sent to the Institute for Fish and Wildlife Health (FIWI) for post-mortem examination and baseline health data collection (Swiss Federal Office of Environment, 2016). During necropsy, intestinal contents from all submitted carcasses are sampled and forwarded to the Institute of Parasitology in Bern for coprological analysis. Consequently, this study provides a reliable estimate of cestode occurrence in the wolf population in Switzerland. However, some carcasses remain undiscovered. The distribution of wolf samples in this study reflects the geographical distribution of resident wolves in Switzerland during the investigation period. In contrast, samples from domestic dogs may be sent to various laboratories. This study primarily includes samples from domestic dogs in western Switzerland and therefore does not represent the entire Swiss dog population. Neither for wolves nor for dogs was a distinct geographic pattern in the distribution of various cestode species observed, as these species were found across multiple cantons without a specific distribution pattern. The wolf samples were indicated on the map at the location where the wolves were shot or found, and dog samples at the postcode of the owner's residence. There are transboundary wolves that could have been infected in neighbouring countries (Graf and Fischer, 2023; KORA - Raubtierökologie und Wildtiermanagement, https://www.kora.ch/de/, accessed 6th June 2024). This also applies to dogs that travel with their owners. The location on the maps is therefore only where the parasite was found and does not correlate with the location of infection.

All samples from free-ranging wolves, as well as samples from nine dogs were collected during necropsy, allowing the macroscopic detection of intestinal parasites and collection of intestinal content samples. This approach increased the chances of finding adult tapeworms. However, in the standard necropsy protocol, the intestines are not completely opened along their full length and some potentially present parasites may have been missed. This could explain why, in seven wolves, taeniid eggs were detected by flotation, but no adult parasites or proglottids were found or recovered during necropsy and macroscopic examination. In contrast, the samples from dogs primarily consisted of faecal samples, which inherently reduces the probability of finding adult worms or proglottids compared to necropsy. Only two out of 16 faecal samples positive for cestode eggs by flotation contained adult worm stages. This study highlights that relying solely on faecal flotation to diagnose cestode infections may lead to an underestimation of prevalence. A significant number of wolves, specifically 34 out of 76 (44.7%), had detectable worm stages during necropsy but tested negative for cestodes in their faeces or intestinal content by flotation. As a result, the flotation technique only detected 55.3% of the infected animals and is therefore not reliable to exclude a cestode infection. This fact was also observed in a previous comparative study in cats, in which *Hydatigera taeniaeformis* stages were found at necropsy in 12 cats, but a positive result by the same flotation method as used in the present study was only observed in 6 out of these cases (Furtado Jost et al., 2023). The discrepancy between the prevalence detected by flotation and overall prevalence can be attributed to several factors. The flotation method may miss infections due to the presence of non-gravid adult worms in the intestines or the uneven distribution of cestode eggs in the faeces (David and Lindquist, 1982; Dryden et al., 2005). Given the small sample amount analysed (5–10 g), it is possible that the sample contains too few or no cestode eggs. Additionally, the eggs of cyclophyllid cestodes (e.g., *Taenia* spp., *Mesocestoides* spp., *Dipylidium* spp.) are most commonly released from the proglottids in the environment rather than within the intestine, which means that very few eggs are found in the faeces (Deplazes et al., 2021). Because we examined the wolves' intestinal contents rather than faecal samples, the probability that the proglottids were still intact and the eggs had not yet been excreted is even higher than in the faecal samples from dogs. It is known, that *Mesocestoides* spp. eggs are detected with less than 0.6% sensitivity by flotation (Széll et al., 2015). This is consistent with this study's findings, as the flotation performed on three wolves and one dog infected with *Mesocestoides* spp. all yielded negative results. It is likely that a greater number of dogs are infected with *Mesocestoides* spp., as these infections can only be detected through the presence of excreted worm stages or those found at necropsy. In some cases, adult worm stages found at necropsy or in faeces might be immature and thus not yet produce eggs, which can lead to negative flotation results. Besides, taeniid eggs are denser and less likely to float in flotation solutions compared to most nematode eggs (David and Lindquist, 1982; Dryden et al., 2005), meaning that faecal analysis alone might yield false-negative results.

*Dibothriocephalus latus* can be diagnosed by macroscopic detection of shed proglottids in faeces or by detection of eggs by the sedimentation method, due to their higher specific gravity than most helminth eggs (Schnieder, 2006; Deplazes et al., 2021). In the present study, all 2065 samples were examined macroscopically and 2063 by flotation, but only 127 were examined by the sedimentation method. *D. latus* was identified macroscopically in only one sample, and all 127 examined sediments were negative for cestodes. The prevalence of *D. latus* is therefore most likely underestimated, as most samples are not analysed by the sedimentation method and *D. latus* eggs may be missed if samples are only analysed by flotation.

This study aimed to detect co-infections with *Taenia* spp. and *Echinococcus* spp., as well as with other cestode species, but deep investigation of co-infections with different *Taenia* species was not a primary objective. Therefore, when both worm stages and eggs were present in a sample, the identification to the species level was performed by sequencing the PCR products derived from worm stages. This approach avoids the sequencing from faecal material due to a potential mixture of different *Taenia* species rendering inconclusive results. Nevertheless, in a random sample of 15 wolves, both PCR products derived from adult worms as well as from faecal samples containing taeniid eggs were sequenced. In twelve cases, the sequencing of both amplicons revealed the same cestode species, in three cases a co-infection of *T. hydatigena* and *T. serialis* could be detected, and in three wolves the sequencing of amplicons from eggs resulted in superimposed signals, which were most probably caused by a mixture of DNA from different cestode species. As the chances of finding a clear co-infection in this way are small, sequencing of amplicons from faecal samples containing taeniid eggs was only performed on samples that did not contain adult stages. In order to reliably diagnose the co-infections, amplicons from each individual adult worm would have to be sequenced, and PCR products from eggs would have to be cloned which was not possible within the scope of our study. It is therefore likely that some animals were infected with more than one cestode species, remaining underdiagnosed. However, samples containing taeniid eggs were analysed by multiplex PCR (Trachsel et al., 2007) to ensure the detection of potential coinfections with *Taenia* spp. (or other cestodes) and *E. granulosus* and/or *E. multilocularis*, as this PCR allows such differentiation.

The prevalence of cestode infection in wolves was 62.8% (95% CI: 53.6–71.4%) in this study, which is comparable to the prevalence of 50.0% (45.0% *Taenia* spp., 5.0% *Mesocestoides* spp.) found in Sweden (Al-Sabi et al., 2018). In Italy, lower prevalence rates than in Switzerland were recorded over several years: 33.0% in northern Italy (Gori et al., 2015), 34.2% (Macchioni et al., 2021) and 43.2% (Crotti et al., 2023) in central Italy. The lower prevalences observed in the studies from northern and central Italy may be explained through their sole reliance on faecal examinations, not including necropsy (Gori et al., 2015; Macchioni et al., 2021; Crotti et al., 2023). These prevalences are in line with the one we obtained only by faecal/intestinal content flotation (34.7%).

In the present study, five cestode species infecting wolves were identified at the species level: *T. hydatigena* (46/121; 38.0%), *T. serialis* (23/121; 19.0%), *M. canislagopodis* (2/121; 1.7%), *T. ovis* (1/121; 0.8%) and *E. multilocularis* (1/121; 0.8%). Wolves carrying *T. hydatigena* have been reported in various European countries including Italy (Gori et al., 2015; Poglayen et al., 2017; Bariselli et al., 2023), Germany (Lesniak et al., 2017; Bindke et al., 2019), Portugal (Guerra et al., 2013), Serbia (Ćirović et al., 2015), Estonia (Moks et al., 2006), and Latvia (Bagrade et al., 2009). The prevalence of *T. hydatigena* ranges from 2.9% (2/69) in Germany (Bindke et al., 2019) to 41.2% (14/34) in Latvia (Bagrade et al., 2009). *T. hydatigena* infects a wide variety of intermediate hosts such as roe deer (Letkova et al., 2008; Jarošová et al., 2022; Bariselli et al., 2023), red and fallow deer (Letkova et al., 2008), chamois (Jarošová et al., 2022), sheep (Corda et al., 2020; Jarošová et al., 2022; Abdollahi et al., 2023) as well as wild boars (Sgroi et al., 2020; Jarošová et al., 2022; Bariselli et al., 2023). The prevalence reported here correlates with the highest prevalence found in Europe (Latvia), where wolves were also investigated by necropsy and macroscopic examination (Bagrade et al., 2009).

*T. serialis* was found in wolves from several countries as well, such as Italy (Bariselli et al., 2023; Crotti et al., 2023), Serbia (Ćirović et al., 2015), Portugal (Guerra et al., 2013) and Germany (Bindke et al., 2019), although in the latter it was not possible to distinguish between *T. serialis* and *T. krabbei*. Traditionally known intermediate hosts for *T. serialis* are hares and rabbits and more rarely rodents (Deplazes et al., 2021). However, recent studies in Italy revealed the European roe deer (*Capreolus capreolus*) as a new intermediate host for *T. serialis* (Morandi et al., 2022; Bariselli et al., 2023). Despite preliminary results indicate that 1.1% of the Swiss wolves prey spectrum is made up of hares, it is possible that *T. serialis* infection could occur due to preying on roe deer, as these represent a major part of their diet in Switzerland (KORA - Raubtierökologie und Wildtiermanagement, https://www.kora.ch/de/, accessed 6th June 2024), thus maintaining the life cycle of *T. serialis* by a different way than previously assumed. These assumptions would also be sustained with the high *T. hydatigena* prevalence already discussed. In addition, dietary analyses from several European countries show that the main food sources of wolves are wild ungulates, with deer (roe deer, red deer, fallow deer) and wild boar being the most common, along with a smaller proportion consisting of small mammals and domestic animals (Milanesi et al., 2012; Newsome et al., 2016; Ståhlberg et al., 2017; Ferretti et al., 2019; Mysłajek et al., 2022; Buzan et al., 2024).

Wolves hosting *T. ovis* seem to be rarer, as they have been reported in few countries and with a lower prevalence than other cestodes: i.e., 2.2% (4/179) in Italy (Gori et al., 2015), 8.8% (3/34) in Latvia (no differentiation between *T. ovis* and *T. krabbei)* (Bagrade et al., 2009) and 15% (4/26) in Estonia (Moks et al., 2006). Despite of our highly sensitive approach, the observed prevalence of *T. ovis* infection is among the lowest reported in Europe. It is known that wolves in Switzerland occasionally prey or scavenge on sheep (Gervasi et al., 2021; Singer et al., 2023; KORA - Raubtierökologie und Wildtiermanagement, https://www.kora.ch/de/, accessed 6th June 2024), allowing the life cycle of this tapeworm to be fulfilled. Herding dogs, however, may also be carriers of cestodes such as *T. hydatigena*, *T. multiceps* or *T. ovis*, and may thus infect sheep as they live in close contact with the herd (DeWolf et al., 2013; Morandi et al., 2020). A parasite prevalence of 23% including 2% taeniid eggs was reported for Swiss herding dogs (Frey et al., 2010). However, national regulations require the removal of carcasses of dead animals, reducing their availability to canids, which may explain the low prevalence of *T. ovis*.

The prevalence of *E. multilocularis* in the sampled wolves in this study was low, in agreement with studies from other European countries. In Latvia (Bagrade et al., 2009) and Germany (Lesniak et al., 2017), only two (5.9%; 2/34) and one (2%; 1/53) wolf respectively were found to be infected with *E. multilocularis*. The fox tapeworm (*E. multilocularis*) is endemic in central Europe, but is expected to spread to northern latitudes and the Alpine region in the future due to global changes (Cenni et al., 2023). *E. multilocularis* has been reported unexpectedly in five wolf faecal samples and in at least two shepherd dogs in the south-western Italian Alps which indicates a spread to the south (Massolo et al., 2018). *E. granulosus*, on the other hand, is more common in Italian wolves and has already been detected in at least four studies (Gori et al., 2015; Poglayen et al., 2017; Macchioni et al., 2021; Crotti et al., 2023). *E. granulosus* has also been observed in wolves in Estonia (Moks et al., 2006), Latvia (Bagrade et al., 2009) and Portugal (Guerra et al., 2013), but currently not in Switzerland.

Wolves infected with *Mesocestoides* spp. have been reported with low prevalence in several countries: Italy (Crotti et al., 2023), Germany (Lesniak et al., 2017), Latvia (Bagrade et al., 2009), Estonia (Moks et al., 2006) and Serbia (Ćirović et al., 2015) with *M. litteratus* and *M. lineatus* as the most common species. In this study, two wolves were found infected with *M. canislagopodis* (sequence match 99.55%). This cestode has so far only been described in Iceland, with arctic foxes (*Vulpes lagopus*) (Skirnisson et al., 1993), domestic dogs and cats as definitive hosts (Skirnisson et al., 2016a). The gyrfalcon (*Falco rusticolus*) was described as a new primary definitive host, but the parasites seem not to be able to mature (Christensen et al., 2015). The first intermediate host for *M. canislagopodis* is still unknown. The rock ptarmigan (*Lagopus muta)* (Skirnisson et al., 2016b) and the wood mouse (*Apodemus sylvaticus*) (Jouet et al., 2023) have been described to harbour tetrathyridia and therefore, to act as intermediate hosts in Iceland. This is the first detection of *M. canislagopodis* outside Iceland, and *Apodemus* spp. may be acting as an intermediate host in Switzerland. Further studies on definitive and intermediate hosts would be needed to confirm these assumptions. Altogether, the identified cestode species suggest that wolves are mostly infected by the predation and consumption of wildlife. In Switzerland, the prevalence of cestode infection in dogs from the present study was low (0.9%). Comparable or higher prevalence rates have been reported in other European countries such as Spain (1.6%) (Mateo et al., 2023), France (<1%) (Bourgoin et al., 2022) and Poland (11.9%) (Karamon et al., 2019). In central and north-eastern Italy, no cestodes at all were found in domestic dogs by coproscopy (La Torre et al., 2018). Previous studies on Swiss dogs found prevalences of cestode infections of 2% (Frey et al., 2010), 1.8% (Sager et al., 2006), 0.8% (faeces from Robidog® containers) and 0.2% (faeces from grassland) (Hauser et al., 2015), which are comparable to the results of this study. *E. multilocularis* is endemic in Switzerland (Torgerson et al., 2010; Deplazes et al., 2017) and owners are therefore encouraged to perform frequent deworming. According to the European Scientific Counsel Companion Animal Parasites (ESCCAP, www.esccap.org, accessed 12th August 2024) it is recommended to deworm domestic dogs at least four times a year or to have their faeces tested for worm eggs and/or worm stages. Depending on the risk of infection, ESCCAP recommends up to 12 deworming treatments per year. Thus, regular deworming of pets may contribute to the low prevalence observed. In Switzerland, there are no stray dogs and dog owners are obliged to properly dispose dog faeces which helps to break the life cycle of parasites (Hauser et al., 2015). In addition, many owners may choose to directly get their pets dewormed if they observed helminths, without sending samples for examination. Samples are more likely to be submitted in case of persistent or recurrent infections or unexplained gastrointestinal issues, potentially leading to an underestimation of true prevalence. As noted in section 4.3, the flotation method seems to be insufficiently sensitive to detect cestode infections, resulting in a falsely low prevalence. This limitation is particularly relevant for dogs, as faecal samples are more frequently analysed than necropsy material, decreasing the possibility of finding worm stages. The 19 dogs in which a cestode infection was diagnosed were all different animals, thus avoiding the possibility of pseudo replication. However, it is possible that a small number of the negative dogs might have been tested on more than one occasion during the specified time period.

The following cestodes have been identified in dogs living in Switzerland: *T. crassiceps* (6/2065; 0.3%), *E. multilocularis* (3/2065; 0.1%), *T. polyacantha* (1/2065; 0.05%), *Mesocestoides* spp. (2/2065; 0.1%) and one case of *Dibothriocephalus latus* (1/2065; 0.05%) infection. These results indicate that dogs become infected with cestodes primarily through the consumption of rodents, as they are the intermediate hosts of *T. crassiceps*, *T. polyacantha* and *E. multilocularis* (Deplazes et al., 2021). It is known that the dog infected with *D. latus* was regularly fed with raw fish caught in a Swiss lake, giving support to a local life cycle. *Mesocestoides* spp. have two intermediate hosts: the first one is not yet known but an arthropod is suspected, while numerous vertebrates such as amphibians, reptiles, birds or mammals were identified as second intermediate hosts (Deplazes et al., 2021). In a former study conducted in Switzerland, seven examined dogs were infected with taeniid worms (7/505; 1.4%) and two with *D. latum* (2/505; 0.4%) (Sager et al., 2006). The taeniid infections were not identified to the species level, which complicates direct comparison with the findings of this study. In the canton of Zurich, Switzerland, a study investigated the contamination of grassland with dog and fox faeces and revealed *T. crassiceps* in both dog and fox faeces (Hauser et al., 2015). This shows that this taeniid species is commonly infecting domestic and wild canids in Switzerland. A recent study from Poland revealed not only quantitatively more cestode infections in dogs but also a higher variability of tapeworm species (Karamon et al., 2019). In that study, species such as *T. crassiceps* (2/268; 0.7%), *E. multilocularis* (1.5%) and *M. litteratus* (3/268; 1.1%) were found in dogs, in agreement with the present study, but also *T. hydatigena* (14/268; 5.2%), *T. taeniaeformis* (3/268; 1.1%), *T. pisiformis* (1/268; 0.4%) and *T. ovis* (1/268; 0.4%) were diagnosed. This reveals differences in the epidemiology of these taeniid species in Poland, as well as on the dietary habits of the dogs, which besides small mammals (as in Swiss dogs), seemed to include ingestion of infected ruminant tissues. *Dipylidium caninum* was found in dogs from Spain (Mateo et al., 2023) and France (Bourgoin et al., 2022), but not in the present study. A possible explanation for the lack of *D*. *caninum* infections in Switzerland could be that flea infestations are treated quickly, thus breaking the infection cycle. Besides, as discussed above, *D. caninum* eggs are rarely present free in the faeces, accounting for false negative results by flotation (Little et al., 2023).

In Europe, human alveolar echinococcosis is considered one of the most pathogenic zoonosis (Kern et al., 2003; Bouwknegt et al., 2018). In this study, *E. multilocularis* was detected in three dogs (3/2065; 0.1%) and one wolf (1/121; 0.8%). Despite the low prevalence, these infected animals pose a potential risk to humans, particularly the infected domestic dogs due to close contact with people. The present study identified *T. crassiceps* as the most frequently diagnosed tapeworm in Swiss dogs (6/2065; 0.3%), consistent with previous studies in Swiss dogs, foxes (Hauser et al., 2015) and one chinchilla (Basso et al., 2014). *T. crassiceps* can lead to human cysticercosis in immunosuppressed (François et al., 1998; Maillard et al., 1998; Heldwein et al., 2006) but also in immunocompetent humans (Arocker-Mettinger et al., 1992; Chuck et al., 1997; Ntoukas et al., 2013). Two cases of human *T. crassiceps*-cysticercosis have been reported in Switzerland (Goesseringer et al., 2011; Schmid et al., 2014). *T. serialis* was the second most common cestode species in Swiss wolves (23/121; 19.0%). Human coenurosis is traditionally attributed to *T. multiceps,* however, rare cases of human coenurosis caused by *T. serialis* affecting the subcutis (Tappe et al., 2016) and the central nervous system (Yamazawa et al., 2020; Labuschagne et al., 2022) have been reported. Almost 40% of the examined wolves in Switzerland were infected with *T. hydatigena*. Although larval infections with this *Taenia* species have been reported in non-human primates, human infections are thought to be extremely rare (Deplazes et al., 2019).

The repopulation of the Eurasian wolf in Switzerland does not seem to represent a threat to our pets with regards to cestode infections. The only two cestode species found in both wolves and dogs were *Mesocestoides* spp. and *E. multilocularis*. However, other wild canids living in Switzerland, such as foxes or golden jackals, have been repeatedly reported to carry *E. multilocularis* with high prevalences, as well as other tapeworms (Brossard et al., 2007; Reperant et al., 2007; Hauser et al., 2015; Muchaamba et al., 2021; Frey et al., 2022) and thus playing a more relevant role in indirect transmission through intermediate hosts to our domestic animals. Nevertheless, wolves can contaminate the environment, since only a few adult tapeworms (e.g. for *T. hydatigena* or *T. ovis*) can excrete countless eggs, which serve as an infection source for wild ruminants and livestock.

Infection of intermediate hosts with cestodes transmitted by canids can make their organs or even the entire carcass unsuitable for human consumption (Deplazes et al., 2021). There are several studies documenting outbreaks of larval cestodosis in livestock such as cysticercosis (*T. ovis*), coenurosis (*T. multiceps*) or cystic echinococcosis (*E. granulosus*), which were caused by infected dogs and resulted in significant economic losses (Schweizer et al., 2006; Eichenberger et al., 2011). Human infections with *D. latus* do occur, but transmission does not result directly from the contact with infected dogs, but through consumption of raw or undercooked fish harbouring larval stages (Deplazes et al., 2021). Consequently, fish from areas inhabited by infected dogs might represent a source for human infection.

## 5 Conclusions

This study provides a first overview of cestode infections in Eurasian wolves and domestic dogs in Switzerland. Wolves are frequently infected with cestodes. Molecular investigations allowed identification of the circulating cestode species in Switzerland, bringing insight into the different infection sources of domestic and wild canids in the region, as well as on the life cycles and potential impact of these parasites on wildlife, livestock, and human health. Wolves seem to become infected mainly through the consumption of ruminants (IH of *T. hydatigena*, *T. serialis* and *T. ovis*) and, to a lesser extent, probably also through small mammals such as hares (IH of *T. serialis*) and rodents (IH of *E. multilocularis*, *T. serialis*, *Mesocestoides* spp.). Dogs became infected with cestodes primarily through consumption of rodents (IH of *T. crassiceps*, *T. polyacantha*, *E. multilocularis* and possibly *Mesocestoides* spp.) or raw fish (*D. latus*). Wolves are a source of environmental contamination with *T. hydatigena* or *T. ovis* eggs which may occasionally infect livestock. Only few dogs were infected with cestodes; however, they still pose a risk to humans as they may carry cestodes like *E. multilocularis* or *T. crassiceps*, which can cause diseases such as alveolar echinococcosis or human cysticercosis. The flotation method failed to detect infection in some wolves as shown by necropsy, suggesting that the actual prevalence in dogs might be higher than observed in our study.

## CRediT authorship contribution statement

**Anna Schneider:** Writing – review & editing, Writing – original draft, Visualization, Methodology, Investigation, Formal analysis, Data curation. **Gastón Moré:** Writing – review & editing, Visualization, Validation, Supervision, Methodology, Data curation. **Mirjam Pewsner:** Writing – review & editing, Supervision, Resources, Investigation. **Caroline F. Frey:** Writing – review & editing, Supervision, Investigation, Funding acquisition, Formal analysis, Data curation, Conceptualization. **Walter Basso:** Writing – review & editing, Validation, Supervision, Methodology, Investigation, Funding acquisition, Formal analysis, Data curation, Conceptualization.

## Funding

The study was made possible by internal funding from the Institute of Parasitology of the 10.13039/100009068University of Bern, Switzerland.

## Declaration of competing interest

All the authors are free from conflict of interests which could potentially bias the present study.
